# Optical Relaxation Time Enhancement in Graphene-Passivated Metal Films

**DOI:** 10.1038/srep30519

**Published:** 2016-07-27

**Authors:** Sunny Chugh, Ruchit Mehta, Mengren Man, Zhihong Chen

**Affiliations:** 1 School of Electrical and Computer Engineering, Purdue University, West Lafayette, IN 47907, United States; 2 Birck Nanotechnology Center, Purdue University, West Lafayette, IN 47907, United States

## Abstract

Due to the small skin depth in metals at optical frequencies, their plasmonic response is strongly dictated by their surface properties. Copper (Cu) is one of the standard materials of choice for plasmonic applications, because of its high conductivity and CMOS compatibility. However, being a chemically active material, it gets easily oxidized when left in ambient environment, causing an inevitable degradation in its plasmonic resonance. Here, for the first time, we report a strong enhancement in the optical relaxation time in Cu by direct growth of few-layer graphene that is shown to act as an excellent passivation layer protecting Cu surface from any deterioration. Spectroscopic ellipsometry measurements reveal a 40–50% reduction in the total scattering rate in Cu itself, which is attributed to an improvement in its surface properties. We also study the impact of graphene quality and show that high quality graphene leads to an even larger improvement in electron scattering rate. These findings are expected to provide a big push towards graphene-protected Cu plasmonics.

Optical properties of noble metals such as copper (Cu), gold (Au) and silver (Ag) have been widely studied for a long time^1^,^2^,^3^,^4^,^5^,^6^,^7^,^8^,^9^,^10^. Metals, in general, have a large density of free electrons, as a result of which, at frequencies roughly below the plasma frequency, most of the light incident on a metal surface is reflected off and the electric and the magnetic fields exponentially decay away from the surface, barring inter-band transitions. Consequently, surface scattering of electrons plays an important role in determining the response of these materials to the high frequency electromagnetic waves and defining their plasmonic characteristics. Cu is one of the most sought after materials for plasmonic applications, due to its low electron scattering rate and compatibility with standard silicon manufacturing processes, namely with complementary metal oxide semiconductor (CMOS) technology, however, it oxidizes very easily in ambient environment resulting in undesired surface degradation. Electrons impinging on the surface of a metal can either undergo specular or diffuse scattering depending on the nature of the local surface states. Diffuse surface scattering can greatly reduce the electron mean free path, a phenomenon that has been very clearly observed in bare Cu due to the formation of Cu oxide^11^. To this end, there is a growing need for thin passivation layers that can isolate the Cu’s surface from corrosive or oxidizing environment and preserve its intrinsic properties. In this regard, graphene, an sp^2^-bonded 2D material, seems to satisfy those requirements since it acts as an excellent passivation layer for Cu^12^,^13^,^14^. However, the influence of directly grown graphene (or other 2D materials) on the optical properties of metallic substrates is still unknown.

In our recent work on graphene-covered Cu nanowires, we showed that coating Cu with few-layer graphene results in lower electrical resistivity and higher thermal conductivity in Cu at DC conditions^14^. Those observations were interpreted as an outcome of reduced surface scattering, however, they were not direct experimental measurements of the scattering rate in Cu itself. In this work, we use spectroscopic ellipsometry to directly probe the impact of few-layer graphene on surface scattering of electrons in Cu and observe a 40–50% decrease in the optical relaxation rate in graphene-covered Cu as compared to Cu with a layer of native Cu oxide. This reduction is a result of improved surface scattering at the Cu-graphene interface, which is attributed to the effect of the graphene passivation layer. Graphene is an sp^2^-bonded low density of states material that interacts very weakly with its underlying substrate, as opposed to native Cu oxide that creates trap states at the Cu–Cu oxide interface resulting in diffuse surface scattering of electrons. To our knowledge, this is the first demonstration that will effectively eliminate one of the key obstacles, i.e. Cu corrosion in air, to facilitate the use of Cu in plasmonic applications. Furthermore, while the focus of this study lies on Cu only, we expect to achieve similar improvement in the surface properties of other metals that are relevant for plasmonics such as Ag, and even recently studied alternative materials such as TiN and TaN.

## Results

### Experimental Design

**Figure 1:**
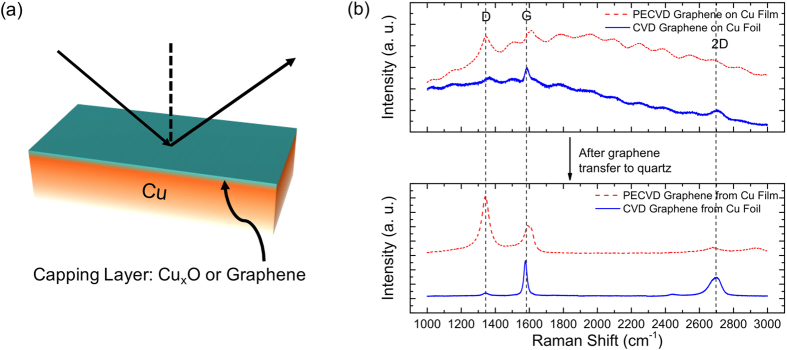
(**a**) Schematic of Cu samples used for ellipsometry studies in this work. Ellipsometry measurements are carried out on both Cu films and Cu foils, each of two different kinds-one covered with native Cu oxide and another with directly grown few-layer graphene. (**b**) Raman spectra of graphene grown on a Cu film at 550 °C using a remote PECVD process (dotted red) and of high-quality graphene grown on a Cu foil at 1035 °C using a CVD process (solid blue), acquired using a 532 nm laser. Spectra are displaced vertically for clarity. Upper panel shows spectra directly acquired on Cu and lower panel shows the corresponding spectra after transferring graphene to a quartz substrate. Graphene’s signature peaks–D, G and 2D–are also labeled. Exact positions of these peaks can be influenced by graphene quality and its substrate. Dotted lines only serve as a guide to the eye.

In this work, we analyze the impact of graphene on electron scattering in Cu by carrying out ellipsometry measurements on two different Cu film samples–one with a few-layer (2–3 layers) graphene coverage (Cu-G) and the other with a layer of Cu oxide coverage (Cu-Cu_x_O) (schematic shown in Fig. 1(a)). Both samples consist of a 450 nm thick Cu film on top of a 5 nm Ta adhesion layer that are deposited by e-beam evaporation on a 20 nm SiO_2_-Si wafer at rates of 0.3 nm/s and 0.1 nm/s, respectively. Cu-G sample is prepared by a remote plasma enhanced chemical vapor deposition (PECVD) process at 550 °C using C_2_H_2_ as the carbon precursor in an Ar gas environment. This process has the advantage of completely reducing the native Cu oxide, followed by conformal graphene growth on the pristine Cu surface. Detailed graphene growth conditions are listed in the Methods section. Cu-Cu_x_O sample is prepared by a similar process, except that the C_2_H_2_ precursor is replaced by H_2_. Similar to the C_2_H_2_/Ar plasma process, any native oxide present before the H_2_/Ar plasma process is completely reduced. However, fresh oxide is immediately formed on the surface when the sample is unloaded from the growth chamber because of the absence of a passivation barrier. The dotted red curves in Fig. 1(b) show the Raman spectra of PECVD graphene directly acquired on Cu film (upper panel) and after transferring it to a 0.5 mm thick quartz substrate (lower panel) (see Methods section for graphene transfer recipe). The signature Raman peaks for graphene, at ~1350 cm^−1^, ~1580 cm^−1^ and ~2700 cm^−1^ are clearly labeled^15^.

The optical response of Cu in the near-IR frequency range (>1000 nm) is mostly associated with the electronic intra-band transitions within the conduction band. As a result, its dielectric constant can be adequately described by the Drude-Sommerfeld free electron model. No critical point corrections are needed as the nearest inter-band transition in Cu occurs at ~500 nm^1^,^2^,^3^. Hence, the complex dielectric constant of Cu, *ε*_*r*_, is given by -


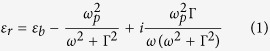


The real and the imaginary parts of the complex dielectric constant are notated by *ε*_*1*_ and *ε*_*2*_, respectively. Here, *ε*_*b*_ is the contribution due to bound electrons and can be roughly approximated as a constant (~6) in the concerned spectral range, and *ω*_*p*_ is the plasma frequency of Cu (~8.7 eV)^1^,^2^,^3^. *Γ*, which is an accurate measure of the scattering rate of electrons (in eV) and is inversely proportional to the electron relaxation time, is the parameter of interest for this study. In the case of a thin film, electron scattering could come from different sources–(1) electron-electron scattering, (2) electron-phonon scattering, (3) lattice defect scattering, (4) grain boundary scattering and (5) surface scattering. (1)–(3) are present in bulk crystalline Cu, while (4) occurs in polycrystalline Cu films and (5) is present because of the small skin depth at optical frequencies.

**Figure 2:**
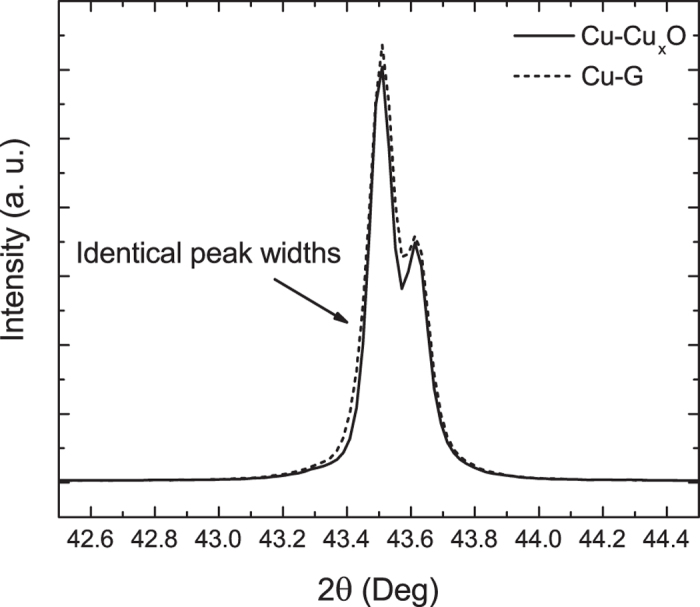
XRD spectra of two different Cu films–Cu-Cu_x_O and Cu-G, showing Cu(111) peak at 43.5°. Samples show almost indistinguishable peak-widths.

Since all process conditions, except using C_2_H_2_ or H_2_, are kept exactly the same for the Cu-Cu_x_O and the Cu-G sample fabrication (including temperature, time, pressure and Ar flow) and the concentration of C_2_H_2_ or H_2_ is relatively insignificant as compared to Ar, we can reasonably assume that the grain size/quality of the Cu-Cu_x_O and Cu-G samples are almost identical. X-Ray Diffraction (XRD) measurements on Cu-Cu_x_O and Cu-G also reveal rather indistinguishable full-width-at-half-maximum values for the Cu(111) peak at 43.5° (Fig. 2), a signature of similar grain sizes of Cu. As a result, background scattering and grain boundary scattering of electrons are assumed to be invariant between these two samples and any difference in electron scattering rate *Γ*, if observed from ellipsometry measurements, can be attributed to different surface scattering of electrons. Difference in surface scattering can potentially arise from varying surface roughness, however, as we found from our AFM study, Cu-Cu_x_O and Cu-G have nearly identical roughness within a grain. Moreover, various studies have shown that small changes in roughness, if any, cannot result in significant changes in surface scattering^8^,^16^. Finally, because *ε*_*b*_ and *ω*_*p*_ are material parameters that shouldn’t vary with surface properties, we expect to obtain identical values from both Cu-Cu_x_O and Cu-G.

### Extraction of the Optical Sheet Conductivity of Graphene

Ellipsometry gives the ratio of the complex reflection coefficients for transverse magnetic and transverse electric polarizations as *r*_*TM*_/*r*_*TE*_ = *tan(Ψ)e*^*−iΔ*^. All ellipsometry measurements described in this work are carried out at 50° using a V-VASE J. A. Woollam Co. ellipsometer. The reflection coefficients described above are a function of dielectric constants and thicknesses of the materials involved. The raw data, available from the measurement in the form of *ψ* and *Δ*, can thus be inverted to obtain the complex dielectric constant of Cu assuming optical properties and thicknesses of other materials in the system are known. As discussed later, these parameters are easily available for the Cu-Cu_x_O material system^17^,^18^,^19^,^20^,^21^. However, for the Cu-G system, we need to obtain the wavelength-dependent optical constants of the few-layer graphene before we can extract those for Cu. In order to do so, we carry out ellipsometry measurements on a graphene-on-quartz sample and use a 2D model to extract the sheet conductivity of graphene, which effectively captures both the complex dielectric constant and the thickness of graphene.

**Figure 3:**
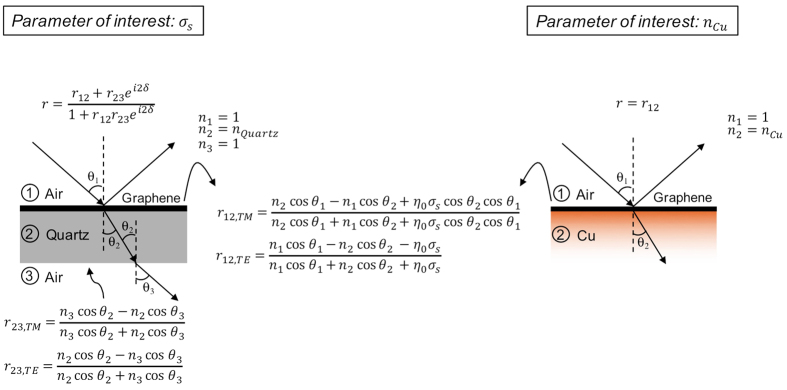
Schematics describing the ellipsometric extraction of *σ*_*s*_ from the graphene-on-quartz sample (left Figure) and *n_Cu_* from the Cu-G sample (right Figure). Both extraction methods employ the same model, with the only difference being that for the graphene on quartz sample, the back side reflection is also considered, while for the graphene on Cu stack, Cu is considered to be semi-infinitely thick.

The left schematic in Fig. 3 elucidates the methodology for the extraction of *σ*_*s*_ from the graphene-on-quartz sample. This sample has two interfaces-the top air/graphene/quartz interface (with reflection coefficient *r*_*12*_) and the bottom quartz/air interface (with reflection coefficient *r*_*23*_), resulting in a total electric field reflection coefficient at the top surface (*r*) of


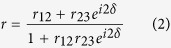


where *δ* is the phase accumulated by the wave in traversing through the quartz substrate and is given by the product of the wavenumber, quartz’s refractive index and its thickness. In our previous work^22^, we have shown that the standard Fresnel reflection coefficients can be modified to incorporate the effect of a surface current arising from graphene by using a 2D model of graphene’s complex sheet conductivity (*σ*_*s*_) to give *r*_*12*_ in the form below -


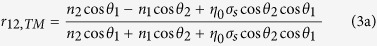



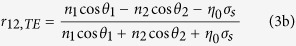


where *n*_*1*_ and *n*_*2*_ are the complex refractive indices of the free space and the quartz substrate, respectively, *η*_*0*_ is the free space impedance and *θ*_*1*_ and *θ*_*2*_ are the angles between the wave vectors and the interface normal in the two regions. *θ*_*1*_ is set to 50° and *θ*_*2*_ can be found using the Snell’s law. *r*_*23*_ takes the form of standard Fresnel reflection coefficients for TM and TE polarizations^23^ and is obtained by taking *σ*_*s*_ = 0 in eq. (3a) and (3b), respectively. The incoherent superposition of light in the transparent quartz substrate is accounted for by using spectrally averaged reflectances that depend on the reflection coefficients in eq. (2). This extraction procedure is described in further detail in our previous work^22^.

**Figure 4:**
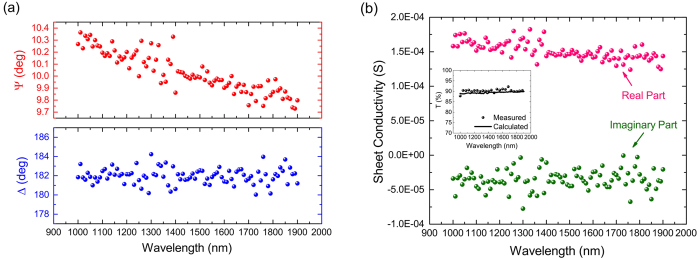
(**a**) Raw ellipsometry data for the graphene-on-quartz sample in the form of *Ψ* and *Δ*. (**b**) Complex sheet conductivity of graphene extracted from the raw data (real part in pink and imaginary part in green). The inset shows the measured (scattered points) and the calculated (solid line) transmission from the sample at normal incidence.

From the experimental *ψ* and *Δ* data (shown in Fig. 4(a)), we extract *σ*_*s*_ of few-layer graphene that is presented in Fig. 4(b). Also shown in the inset is the measured transmittance of the graphene-on-quartz sample at normal incidence as a function of the wavelength, as well as the calculated transmittance from the extracted sheet conductivity data. Measurement is done using a Lambda950 Perkin Elmer spectrophotometer and calculation is based on the method introduced by F. Abelés^22^,^23^. A good agreement between the two validates our extraction of the sheet conductivity of graphene.

### Calculation of the Complex Dielectric Constant of Cu Film Samples

**Figure 5:**
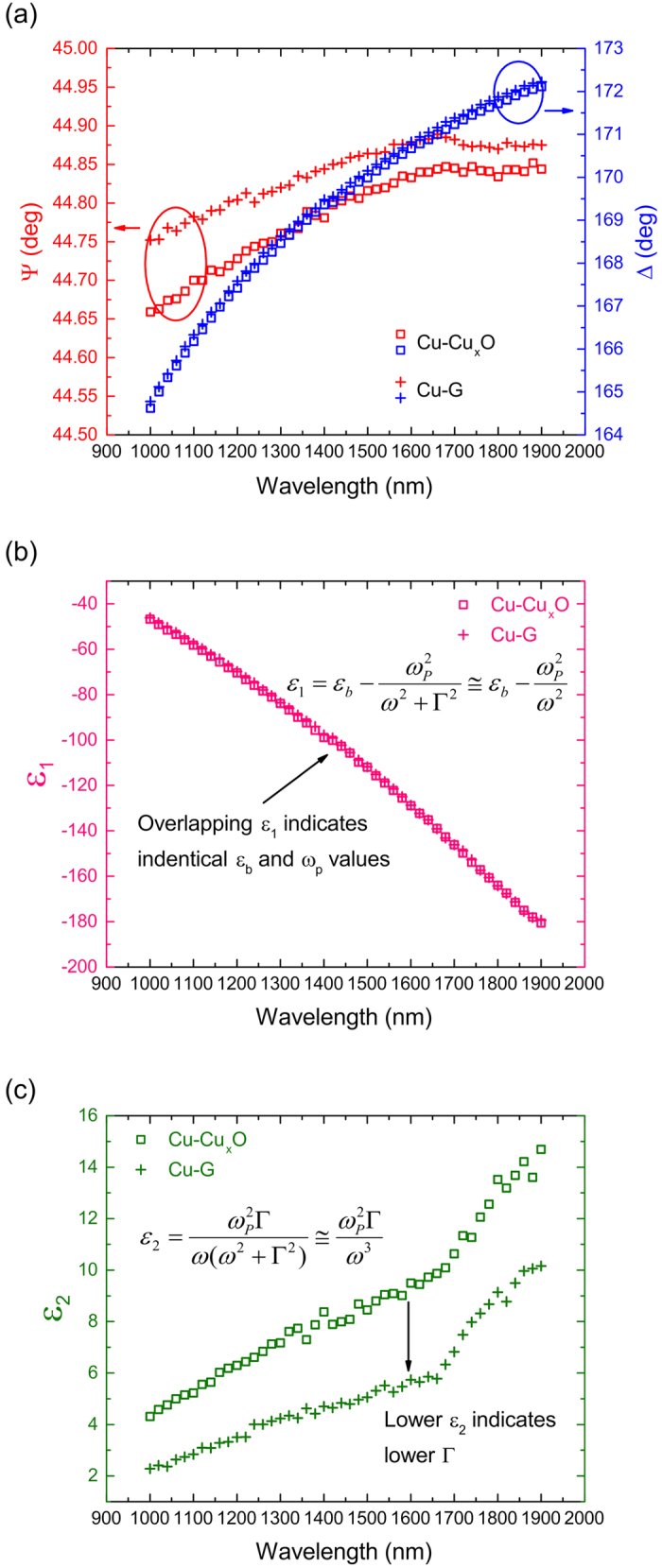
(**a**) Raw ellipsometry data from Cu-Cu_x_O and Cu-G samples (prepared from Cu film) in the form of *Ψ* and *Δ*. (**b**) Real part and (**c**) imaginary part of the complex dielectric constant of Cu as a function of the wavelength, as found from the analysis of Cu-Cu_x_O and Cu-G.

Similar ellipsometry measurements are carried out on Cu-Cu_x_O and Cu-G samples and the raw data are shown in Fig. 5(a). Given that the film thickness of 450 nm is far greater than the skin depth of Cu in the near-IR frequency range (~20 nm), we assume bulk Cu for our ellipsometry analysis. It is well established in literature that the first oxide forming on Cu is Cu_2_O^17^,^18^,^19^,^20^,^21^. Since the ellipsometry measurements are carried out shortly after the sample fabrication, we use a classical bilayer model assuming a very small Cu_2_O thickness (1 nm) for the analysis of the Cu-Cu_x_O sample. However, it should be noted that slightly altering the oxide thickness doesn’t cause any significant change in the final results due to its high optical transparency in the studied wavelength range. Similarly, replacing Cu_2_O with CuO in the bilayer model doesn’t impact our analysis either due to their similar optical constants. Based on these assumptions, we directly invert the raw data and extract *ε*_*1*_ and *ε*_*2*_ of Cu using the pre-installed WVASE32 software package in the ellipsometer. These are plotted in Fig. 5(b,c) respectively.

For the analysis of the Cu-G sample, we use the same sheet conductivity model for graphene, as that of the graphene-on-quartz sample. However, since Cu is completely opaque and can be treated as semi-infinitely thick, the total electric field reflection coefficient for the Cu-G sample is given by eq. (3a) and (3b) for TM and TE polarizations, respectively. In other words, *r*_*23*_* = 0* and *r = r*_*12*_, with *n*_*2*_ being the complex refractive index of Cu in this case and the quantity of interest for this measurement, as shown in the right schematic in Fig. 3. Since *σ*_*s*_ is known from the earlier graphene-on-quartz measurement, *n*_*2*_ can be readily calculated from experimentally obtained *ψ* and *Δ*, which is then used to obtain the dielectric function of Cu by following the relation *ε*_*r*_ = *ε*_*1*_ + *ε*_*2*_ = (*n*_*2*_)^2^. The extracted data of *ε*_*1*_and *ε*_*2*_ are presented in Fig. 5(b,c), respectively. The data for both samples, Cu-Cu_x_O and Cu-G, are then fitted to the Drude model as shown in eq. (1). Values of *ε*_*b*_and *ω*_*p*_ for Cu are found to be 5.9 and 9.0 eV, respectively, for both Cu-Cu_x_O and Cu-G, which are very close to reported values in literature^1^,^2^,^3^. Because *ε*_*b*_and *ω*_*p*_ of Cu are not expected to change between Cu-Cu_x_O and Cu-G samples, and *ε*_*1*_ has a very weak dependence on *Γ*, an overlap of the two *ε*_*1*_ curves is expected and indeed observed in Fig. 5(b), which validates our extraction model based on coefficients in eq. (3a) and (3b). On the other hand, *ε*_*2*_values are markedly different between the two samples. For the same *ω*_*p*_, lower *ε*_*2*_ of Cu in the Cu-G sample indicates a lower *Γ* in Cu. Fitting a frequency independent *Γ* gives *Γ*  = 0.060 eV for the Cu-Cu_x_O sample and 0.037 eV for the Cu-G sample.

In the ellipsometry analysis described above, we have used *σ*_*s*_ of graphene after transfer for the extraction of Cu dielectric constant from the Cu-G sample. However, the *σ*_*s*_ value can change due to transfer and substrate. In order to check the robustness of our extraction, we carry out a sensitivity analysis to find out how the extracted *Γ* of Cu varies on altering *σ*_*s*_. The effect of changing the real and imaginary parts of *σ*_*s*_ is studied by changing one at a time (Table 1). We find that the value of *Γ* is not very sensitive to Im(*σ*_*s*_). When changing it to 0 while keeping Re(*σ*_*s*_) the same, *Γ* varies from 0.037 eV to 0.0362 eV. Similarly, varying Re(*σ*_*s*_) by ±15% results in a small change of *Γ* from 0.0359 eV to 0.0381 eV. As we can see, the extracted Cu parameters are not very sensitive to the sheet conductivity of graphene, hence suggesting that using *σ*_*s*_ of the transferred graphene for the analysis of Cu-G sample is reasonable.

### Calculation of the Complex Dielectric Constant of Cu Foil Samples

**Figure 6:**
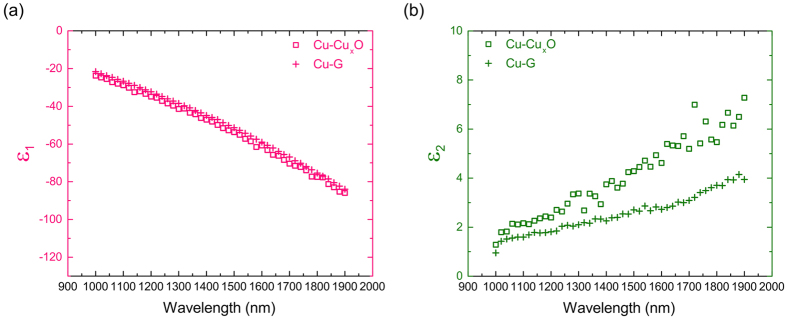
(**a**) Real part and (**b**) imaginary part of the complex dielectric constant of Cu as a function of wavelength, as found from analysis of Cu-Cu_x_O and Cu-G samples prepared from Cu foil.

Raman spectrum of the PECVD grown graphene (Fig. 1(b)) has a high D peak that corresponds to a high defect density in the graphene film. To test whether the quality of the graphene coating is critical for improving the scattering rate in Cu or not, high quality graphene grown on a Cu foil at 1035 °C using a conventional CVD technique (see Methods section) is tested and analyzed using the same method. High temperature CVD grown graphene is almost defect free (as shown by its Raman spectrum in Fig. 1(b)) and the graphene coverage is complete. A control sample is also fabricated by etching graphene in mild O_2_ plasma at 20 W for 15 sec. However, because the O_2_ plasma treatment can also lead to the Cu(OH)_2_ formation^14^, we carefully remove the grown Cu-oxide/hydroxide by remote H_2_ plasma at 100 °C to allow the subsequent formation of fresh Cu_2_O in ambient environment, similar to the one on the Cu film sample. Since Cu foil has a substantially rough surface to begin with, these plasma processes are not expected to cause further surface damage that worsens the surface quality. Furthermore, the obtained values of Drude parameters for the Cu foil samples are expected to be lower than those for the Cu film, because the large surface roughness of the Cu foil causes the depolarization of light^24^. Consistent with the thin film measurement and analysis described above, the ellipsometry extraction for the foil samples yields an excellent overlap of the *ε*_*1*_ plots for the Cu-Cu_x_O and the Cu-G samples and *ε*_*2*_ is significantly lower for Cu-G (Fig. 6). Extracted values of effective Drude parameters-*ε*_*b*,*eff*_ and *ω*_*p*,*eff*_ -of Cu are found to be ~2–3 and ~6.1–6.2 eV, respectively, from both Cu-Cu_x_O and Cu-G samples. A significant sample to sample variation is observed for Cu foil samples due to its relatively wavy surface. Fitting a frequency independent *Γ* yields values of 0.068 eV for Cu-Cu_x_O and 0.032 eV for Cu-G. These values are also tabulated in Table 2. At this point, it becomes imperative to point out that while depolarization causes a significant variation in effective *ε*_*b*_ and *ω*_*p*_, scattering rate (*Γ* ) has been shown to be insensitive to roughness modeling, thus signifying that the extracted numbers of scattering rate represent their true values.

## Discussion

As discussed earlier, reduction in the electron scattering rate can only be attributed to the improved surface scattering because other scattering mechanisms should remain unchanged between Cu-Cu_x_O and Cu-G. This leads us to believe that graphene increases the degree of specularity of electron surface scattering in Cu, as opposed to native oxide on Cu that results in completely diffuse scattering. Such improvement is expected because graphene is believed to interact weakly with the Cu surface states^25^. Measurements on varying the graphene quality seem to evidence that high quality graphene gives rise to a larger improvement in surface properties of Cu, as indicated by a decrease of 0.036 eV in the scattering rate in the graphene-covered Cu foil, in comparison to 0.023 eV decrease in the film covered by PECVD graphene. It is worthwhile noting that although these measurements are done on Cu only, graphene is an excellent protective layer for many other metallic substrates and enhancement in the surface properties of other metal films that can get corroded in ambient environment, e.g. Ag, TiN, TaN, is also expected. Our particular interest in using Cu for this study stems from the fact that one of the biggest hindrances in utilizing Cu as a plasmonic material is its corrosion in air. Recent work on graphene-covered Cu and Ag plasmonics shows that properties of Cu and Ag can indeed be improved by protecting them with graphene^26^,^27^. These studies were however done by transferring graphene onto Cu/Ag and we believe that directly grown graphene, as used in our work, should provide a better metal-graphene interface. Moreover, Cu is the material of choice for interconnects and our work on graphene-covered Cu could promote further research into the investigation of graphene as an alternative barrier material in integrated circuits^28^,^29^,^30^,^31^ to improve Cu’s conductivity. Similar improvement is expected in graphene-covered metallic nanowires that may be used as transparent electrodes for flexible electronic devices^32^,^33^,^34^.

Finally, long-term stability of graphene-protected Cu is of critical importance for any practical applications. To this end, it was shown that while single-layer graphene acts as an excellent passivation layer for short time scales, oxidation of Cu in ambient conditions is accelerated in long-term, initiated through the defects in graphene layer, resulting in severe discoloration of Cu with time^35^. On the other hand, a recent work shows that few-layer graphene should strongly suppress this effect since alignment of defects in the top graphene layer and underlying bottom layers is highly unlikely^30^. We have also observed that our Cu-G samples with 2–3 layers of graphene don’t show any color change even after being left in ambient conditions for more than 6 months. In fact, because a slightly thicker graphene passivation layer (which can be easily achieved through the non self-limiting PECVD process) should lead to the same improvement in Cu’s surface properties, we believe that using few-layer graphene is ideal for long-term stability of such devices. Surface plasmon resonance measurements using transferred graphene on Cu has shown that the plasmonic resonance in graphene-protected Cu doesn’t deteriorate even after several months of exposure to ambient conditions^27^. Graphene is indeed expected to cause a shift in the plasmonic peak position. However, since surface plasmon resonance fields are excited perpendicular to the metal-graphene interface and don’t generate strong surface currents in graphene, we don’t expect any significant degradation in the plasmonic resonance quality^27^.

## Methods

### PECVD Graphene Growth on Cu Film

Cu film sample is cleaned using a triple sonication wash in toluene, acetone and iso-propyl alcohol for 5 minutes each prior to any graphene growth. Sample with graphene (Cu-G) is prepared by a remote-plasma enhanced chemical vapor deposition (r-PECVD) process at 550 °C using a C_2_H_2_ carbon precursor in a commercially available EasyTube 3000 First Nano system with loadlock capability. The cleaned sample is first loaded into the growth chamber loadlock and after evacuating the system to its base pressure of 50 mTorr, the furnace is heated to 550 °C. When temperature is reached, 100 sccm Ar flow is switched on while maintaining a pressure of 130 mTorr and the sample is transferred into the hot furnace zone. 5 sccm of C_2_H_2_ is then introduced that increases the total pressure to 140 mTorr. Keeping flow rates constant, total pressure in the system is increased to 300 mTorr and graphene growth is carried out for 60 min at a plasma power of 500 W that gives 2–3 layer graphene, after which the sample is taken out of the hot zone and then unloaded at room temperature. As described in our previous work on PECVD graphene growth^13^, any native oxide present on Cu is quickly reduced before graphene growth takes place due to the presence of H radicals in plasma environment. This is established by heating a Cu sample in ambient environment at 200 °C to grow thick Cu oxide that changes its color from shiny orange to dark orange and then taking it through the same C_2_H_2_/Ar plasma process. The Cu film retrieves its original color after the process, indicating that the radical environment is strongly reducing. Since graphene acts as an excellent passivation barrier^12^,^13^, it prevents the formation of Cu oxide. This is verified by heating the graphene-on-Cu sample at 200 °C in ambient environment and observing any possible change in color due to Cu oxide formation. No such change in color indicates that Cu is completely covered with graphene that prevents oxygen to diffuse through it, even at elevated temperatures. Consequently, in this work, we refer to this sample as Cu-G.

### Graphene Transfer Process

Graphene transfer to quartz substrate (SPI# 01016T-AB) is achieved by protecting the Cu-G sample with a 200 nm thick PMMA layer and etching away Cu in 1M FeCl_3_ solution, followed by transferring the remaining graphene-PMMA stack onto quartz and finally stripping off PMMA in hot acetone bath at 50 °C for 2 hours^36^,^37^.

### CVD Graphene Growth on Cu Foil

Prior to any graphene growth, Cu foil is cleaned using the same procedure as followed for Cu film. Sample with graphene (Cu-G) is prepared by a thermal chemical vapor deposition (CVD) process at 1035 °C using a CH_4_ carbon precursor. The cleaned sample is loaded into the growth chamber loadlock and after evacuating the system to its base pressure of 50 mTorr, the furnace is heated to 1035 °C. The sample is transferred into the hot furnace zone when temperature is reached. Native oxide on Cu is first reduced by introducing 20 sccm of H_2_ at a total pressure of 500 mTorr for 30 minutes. Graphene growth is initiated by switching on 200 sccm of CH_4_ and reducing H_2_ flow to 12 sccm while keeping the total pressure at 500 mTorr for 10 minutes. Sample is then cooled down slowly while maintaining gas flows and finally unloaded at room temperature. Etching of native oxide and complete coverage of graphene is established using the same method as described above for Cu film. Cu-Cu_x_O sample is prepared by etching graphene on Cu-G sample in a 20 W O_2_ plasma for 10 sec in a commercial PLASMAtech system. The same sample is further taken through a remote Ar (100 sccm) +H_2_ (5 sccm) plasma in EasyTube 3000 First Nano system at 100 °C, 300 mTorr for 15 min with 500 W plasma power to etch any Cu hydroxide/oxide.

## Additional Information

**How to cite this article**: Chugh, S. *et al*. Optical Relaxation Time Enhancement in Graphene-Passivated Metal Films. *Sci. Rep.* **6**, 30519; doi: 10.1038/srep30519 (2016).

## Figures and Tables

**Table 1:** Results from the sensitivity analysis to study the impact of *σ*_s_ on *Γ* in Cu-G sample.

Sheet Conductivity	*Γ* (in eV)
Re(*σ*_*s*_) + i*Im(*σ*_*s*_)	0.037
Re(*σ*_*s*_)	0.0362
1.15Re(*σ*_*s*_) + i*Im(*σ*_*s*_)	0.0359
0.85Re(*σ*_*s*_) + i*Im(*σ*_*s*_)	0.0381

**Table 2:** Drude model parameters for Cu extracted from ellipsometry analysis of Cu-Cu_x_O and Cu-G samples, fabricated on both Cu film and Cu foil.

Cu Film		Cu Foil	
Cu-Cu_x_O	Cu-G	Cu-Cu_x_O	Cu-G
*Γ* = 0.060 eV	*Γ* = 0.037 eV	*Γ* = 0.068 eV	*Γ* = 0.032 eV
Decrease in *Γ* by 0.023 eV		Decrease in *Γ* by 0.036 eV	
*ε*_*b*_ = 5.9		*ε*_*b,eff*_ = 2.0–3.0	
*ω*_*p*_ = 9.0 eV		*ω*_*p,eff*_ = 6.1–6.2 eV	

Values of *ε*_b_ and *ω*_p_ of Cu are equal for Cu-Cu_x_O and Cu-G, while *Γ* shows a ~40% and ~50% decrease from Cu-Cu_x_O to Cu-G, for film and foil, respectively.
